# A randomized trial of decision support for tobacco dependence treatment in an inpatient electronic medical record: clinical results

**DOI:** 10.1186/s13012-019-0856-8

**Published:** 2019-01-22

**Authors:** Steven L. Bernstein, June Weiss, Michelle DeWitt, Jeanette M. Tetrault, Allen L. Hsiao, James Dziura, Scott Sussman, Ted Miller, Kelly Carpenter, Patrick O’Connor, Benjamin Toll

**Affiliations:** 1 Department of Emergency Medicine, Yale School of Medicine, 464 Congress Ave., Suite 260, New Haven, CT 06519 USA; 2 Department of Pediatrics, Yale School of Medicine, New Haven, USA; 3 Department of Medicine, Yale School of Medicine, New Haven, USA; 4 Department of Psychiatry, Yale School of Medicine, New Haven, USA; 5 Yale Cancer Center, New Haven, USA; 6 Optum Center for Wellbeing Research (formerly Alere Wellbeing), Seattle, WA USA; 7 Yale-New Haven Hospital, New Haven, CT USA; 8 Yale Center for Implementation Science, New Haven, CT USA; 9 Pacific Institute for Research and Evaluation, Calverton, MD USA; 10 Department of Public Health Sciences, Medical University of South Carolina, Charleston, SC USA

**Keywords:** Smoking cessation, Tobacco dependence treatment, Decision support, Electronic health records

## Abstract

**Background:**

Smokers usually abstain from tobacco while hospitalized but relapse after discharge. Inpatient interventions may encourage sustained quitting. We previously demonstrated that a decision support tool embedded in an electronic health record (EHR) improved physicians’ treatment of hospitalized smokers. This report describes the effect on quit rates of this decision support tool and order set for hospitalized smokers.

**Methods:**

In a single hospital system, 254 physicians were randomized 1:1 to receive a decision support tool and order set, embedded in the EHR. When an adult patient was admitted to a medical service, an electronic alert appeared if current smoking was recorded in the EHR. For physicians receiving the intervention, the alert linked to an order set for tobacco treatment medications and electronic referral to the state tobacco quitline. Additionally, “Tobacco Use Disorder” was added to the patient’s problem list, and a secure message was sent to the patient’s primary care provider (PCP). In the control arm, no alert appeared. Patients were contacted by phone at 1, 6, and 12 months; those reporting tobacco abstinence at 12 months were asked to return to measure exhaled carbon monoxide. Generalized estimating equations were used to model the data.

**Results:**

From 2013 to 2016, the alert fired for 10,939 patients (5391 intervention, 5548 control). Compared to control physicians, intervention physicians were more likely to order tobacco treatment medication, populate the problem list with tobacco use disorder, refer to the quitline, and notify the patient’s PCP. In a subset of 1044 patients recruited for intensive follow-up, one-year quit rates for intervention and control patients were, respectively, 11.5% and 11.6%, (*p* = 0.94), after controlling for age, sex, race, ethnicity, and insurance. Similarly, there were no differences in 1- and 6-month quit rates.

**Conclusions:**

Although we were able to improve processes of care, long-term tobacco quit rates were unchanged. This likely reflects, in part, the need for sustained quitting interventions, and higher-than-expected quit rates in controls. Future enhancements should improve prescription of medications for smoking cessation at discharge, engagement of primary care providers, and perhaps direct engagement of patients in a more longitudinal approach.

**Trial registration:**

ClinicalTrials.gov, NCT01691105. Registered on September 12, 2012

## Background

Hospitals are smoke-free environments. Hence, individuals who smoke and are admitted undergo a forced period of abstinence. This offers hospitals an opportunity to identify smokers, engage them in treatment for tobacco use disorder, and extend that treatment after discharge. Tobacco’s enduring status as the leading cause of preventable death and illness in the United States has led to screening and treatment being publicly reported standards of the quality of inpatient care, used by the Centers for Medicare and Medicaid Services (CMS) for patients admitted with acute myocardial infarction, pneumonia, or congestive heart failure. It is a core measure of the National Quality Forum and part of an optional measure set offered by the Joint Commission [1].

Further, the recording of tobacco use was identified as an early indicator of meaningful use of electronic health records by the Health Information Technology for Economic and Clinical Health (HITECH) Act [2]. Electronic health records (EHRs) can offer multiple functionalities to help clinicians treat smokers: prompts to offer medication, automated referrals to a tobacco use disorder treatment services, and electronic referral to a tobacco telephone quitline. Most of the published research assessing the efficacy of EHRs in treating smokers focuses on outpatient care, rather than hospitalized smokers. One study of a closed-loop, bidirectional electronic referral to the state tobacco quitline, conducted in two primary care clinics in a single healthcare system, found that the proportion of smokers referred increased from 0.3% to 14% after the e-referral was implemented [3]. Changes in tobacco abstinence were not reported.

A 2012 Cochrane meta-analysis found that tobacco use disorder treatment initiated during hospitalization leads to sustained abstinence only if medication or counseling (or both) treatment continue at least 30 days after hospital discharge [4]. Many of the studies in this meta-analysis provided ongoing care in the form of multiple visits during the hospitalization and post-discharge contacts via telephone or in-person counseling. In most cases, the intervention was delivered by a research nurse or trained tobacco dependence counselor. In most studies, pharmacotherapy in the form of nicotine replacement medicines, bupropion, or varenicline was provided to the patient. The generalizability of these approaches is likely limited, insofar as most hospitals do not have dedicated “tobacco teams” to perform inpatient interventions.

Subsequent clinical trials of tobacco dependence treatment for hospitalized smokers have also used more intense approaches, with mixed results. Eisenberg et al. randomized 302 adult smokers hospitalized with acute coronary syndrome to receive either 12 weeks of varenicline plus six counseling sessions (phone or in-person) or counseling only [5]. The varenicline group had a higher biochemically verified point prevalence abstinence at 24 weeks compared to controls: 47.3% vs. 35.8%, *p* = 0.012. Rigotti et al. randomized 1357 daily smokers to Usual Care or Sustained Care, which consisted of 30 days of tobacco pharmacotherapy, refillable twice, and up to five automated interactive voice response (IVR) phone calls [6]. At 6 months, there was no difference in biochemically verified cessation between Sustained Care and Usual Care arms, 17% vs. 16%, *p* = 0.58. Sherman et al. randomized 1618 lower socioeconomic status smokers to either a faxed referral to the state smokers’ quitline or an intensive counseling arm in which trained masters-level counselors held up to seven post-discharge phone sessions with patients, who were also eligible for 8 weeks of NRT [7]. Self-reported abstinence at 6 months was greater in the intensive counseling arm, 37.4% vs. 31%, RR 1.19 (95% CI 1.01, 1.40).

Our goal was to develop and test a multicomponent tobacco dependence treatment intervention that would not rely on research staff, standing tobacco treatment teams, or provision of study-related drugs to patients. We wished to test the efficacy of a scalable, pragmatic intervention that incorporated intervention components that could extend tobacco dependence treatment beyond the 30-day post-discharge period. We also wanted the intervention to respect clinical workflows typically found on inpatient units.

We have previously reported on the ability of the Electronic Support Tool and Orders for the Prevention of Smoking (E-STOPS) to enhance providers’ treatment of adult smokers admitted to medical services of an acute-care hospital [8]. In this paper, we report the efficacy of E-STOPS on short- and long-term rates of tobacco abstinence.

## Methods

The study was approved by the institution’s Human Investigation Committee, and was registered on September 12, 2012, at www.ClinicalTrials.gov (NCT01691105, https://www.clinicaltrials.gov/ct2/show/NCT01691105?cond=Smoking+Cessation&cntry=US&state=US%3ACT&city=new+haven&draw=2&rank=24).

Study methods have been described in detail previously [8]. This was a two-arm prospective clinical trial, with two groups of subjects—physicians and patients. Randomization occurred at the level of the physician. The primary endpoint was biochemically verified tobacco cessation among patients, performed via in-person carbon monoxide breath testing 12 months after enrollment, for subjects self-reporting tobacco abstinence by phone. Secondary endpoints included self-reported tobacco abstinence at 1 and 6 months. The key secondary endpoint was the provision of tobacco dependence treatment by physicians. Using Curran’s taxonomy for study designs assessing both implementation and clinical effectiveness, this was a Type I design: effectiveness was the primary endpoint, but data assessing important implementation measures are collected and reported [9].

### Contextual analysis for the intervention

Prior to implementation, the study team interviewed providers, administrators, nurses, IT leadership, pharmacists, and individuals external to the hospital to better understand the context in which the intervention would occur. In addition, we reviewed current national developments in policies, law, and regulation regarding information technology and tobacco dependence treatment. Our analysis of context is informed by a template developed by Stange and Glasgow [10]. Details are provided in Table 5.

**Fig. 1:**
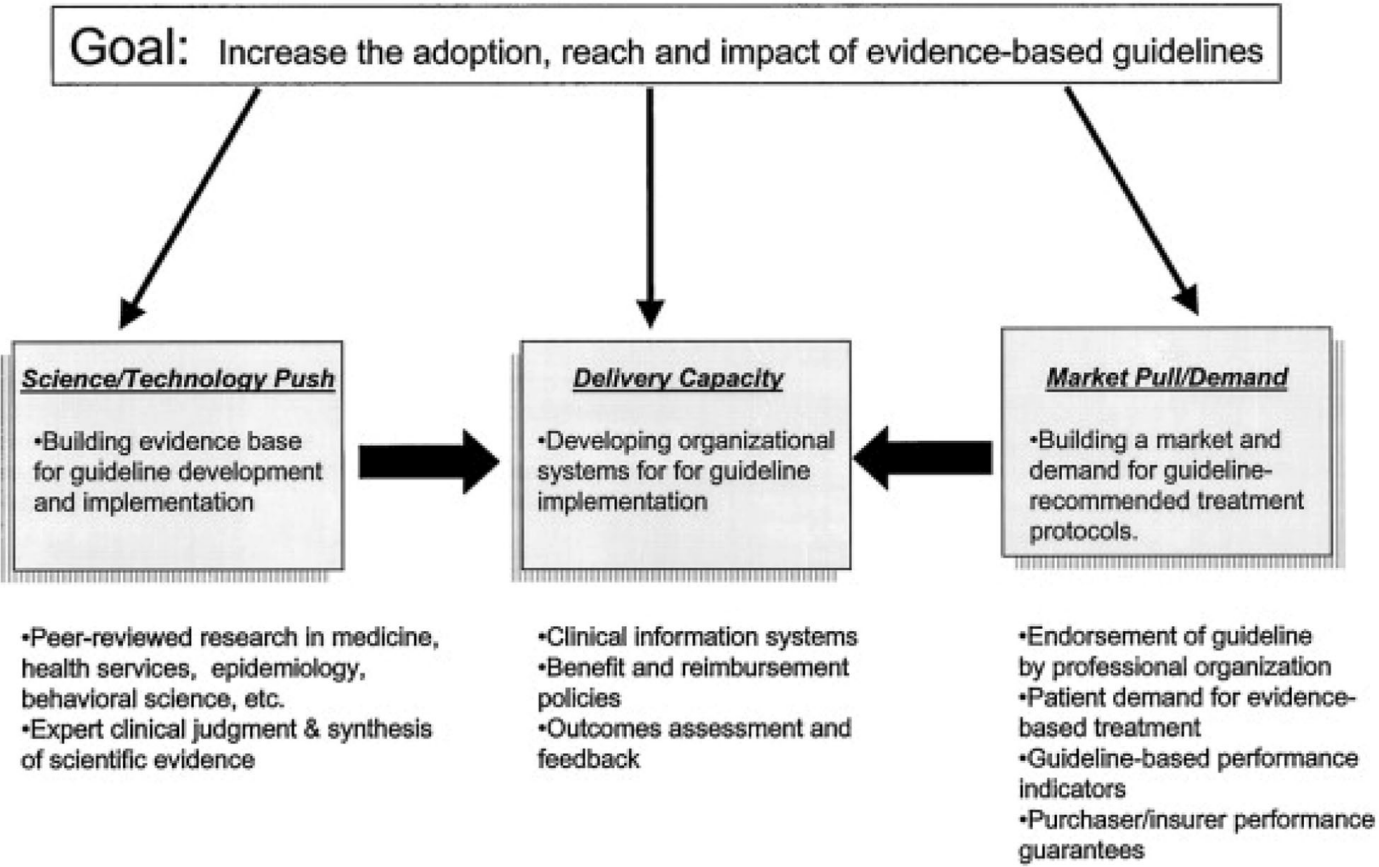
Conceptual model of change. Reproduced from Curry et al. [11]

Curry [11] provided a model to understand the synergies that may facilitate guideline implementation within organizations (Fig. 1). As depicted, “push” factors based on evidence (e.g. Public Health Service guidelines) interact with “pull” factors such as guideline endorsement or regulatory requirements (in this case, CMS and Joint Commission core measures, HITECH “meaningful use” provisions [12]) to encourage organizations to expand delivery capacity. We proposed to hospital leadership to use HIT, outcomes assessment, and feedback, both noted by Curry as means to enhance delivery capacity.

The interventions chosen were designed to facilitate the provision of tobacco dependence treatment to patient participants both during, and after, hospitalization, in accordance with the findings of the Cochrane review. Thus, we added the electronic referral to the state smokers’ quitline, as quitlines have been shown to assist cessation [13, 14]. Nicotine replacement medicines were included in the order set, because of the voluminous evidence base supporting their effectiveness [15]. And adding “Tobacco Use Disorder” to the problem list, as well as emailing the primary care provider, was included because of the evidence supporting the efficacy of provider advice [16], as well as the common need to provide ongoing care for tobacco dependence, a chronic, relapsing condition [17].

E-STOPS consists of several components. The first is a medico-logic module alert built into the EHR that appears on the chart for the first 24 h of the patient’s stay, informing the physician that the patient is a smoker, based upon recorded smoking status in the EHR. The alert has an icon that, when clicked, transfers the provider to a tobacco treatment order set.

Of note, we designed E-STOPS so that tobacco treatment would be the default option. E-STOPS has three functions that were pre-checked, if the alert was accepted. These included (1) an electronic referral to the state’s tobacco quitline, (2) opening of an order set, and (3) adding “Tobacco Use Disorder” to the patient’s problem list. An additional function, an EMR-embedded secure message, was sent to patients’ primary care providers informing them that tobacco dependence treatment was begun and was triggered regardless of whether the physician accepted the alert. Control arm providers were able to execute these functions individually, without the order set, if they sought out each individual function. We elected to have physicians in the control arm treat smokers as usual, without offering additional support via the EHR, in order to best reflect the real-world nature of their practice. Insofar as our hospital does not have a dedicated “tobacco team” available for consultations, we felt this was the optimal approach.

The order set included most FDA-approved medications including nicotine patch, nicotine gum, nicotine lozenge, and bupropion, in addition to a physician reminder that an electronic referral had been made to the state quitline. (Varenicline, which was not on the hospital’s inpatient formulary, was excluded.) All components of E-STOPS were consistent with federal guidelines for the treatment of tobacco use disorder [15].

Physicians, consisting of hospitalists and residents training in internal medicine and emergency medicine, were recruited between August 2013 and July 2014. All physicians enrolled in the study received a brief lecture about the health risks and treatment of tobacco use disorder and gave written informed consent. Randomization of physicians was performed using a random number generator (www.randomization.com) with 1:1 randomization and stratification by specialty. Physicians randomized to the intervention received a brief talk describing the functionality and use of E-STOPS.

E-STOPS was programmed to appear on the charts of adult smokers, at least 18 years of age, admitted to general adult medical units of the hospital. Patients admitted to pediatric, psychiatric, obstetric, or surgical units were not eligible. In addition, eligible participants needed to be residents of New Haven County; provide two collateral contacts; not be currently enrolled in another clinical trial; not be currently using tobacco dependence treatment medications; not be pregnant, nursing, or trying to conceive; own a telephone; and willing to provide written informed consent.

Patients were recruited 7 days/week by research assistants (RAs); written informed consent was given by patients to permit follow-up after discharge. Patients treated by E-STOPS physicians were assigned to the intervention for the index admission and subsequent admissions. Patients treated by control physicians were assigned to the control for the index admission and subsequent admissions.

Physicians randomized to E-STOPS received quarterly feedback reports, informing them how often they ordered medications, referred to the quitline, notified the primary care provider (PCP), and populated the problem list with tobacco use disorder. Individual performance was compared to that of other providers.

Of note, several methods were employed to minimize the risk of contamination, so that patients treated by physicians in the control arm were not “exposed” to E-STOPS, should an E-STOPS physician take over their care. Specifically, E-STOPS was suppressed for all future clinical encounters once the chart was opened by a control physician. This was true during the index admission and for any subsequent readmissions. Conversely, if a patient was first treated by an E-STOPS physician, the prompt and order set would fire for subsequent readmissions.

### Analytic plan

Clinical trial data were recorded in FileMaker Pro and were imported into SAS 9.4. Baseline data are reported with means, standard deviations, medians, and interquartile ranges as appropriate for parametric and nonparametric data. All statistical tests are two-sided, with alpha set at 0.05.

Use of E-STOPS components was evaluated using logistic regression with generalized estimating equations to accommodate the clustering of patients by physician. Analyses are per patient, rather than per admission, to reflect each patient’s exposure to intervention components.

For primary analysis, participants who were lost to follow-up were imputed to be smoking, as is the custom. Comparison of the primary outcome of biochemically confirmed abstinence was performed crudely using chi-square analysis and then by logistic regression with generalized estimating equations (GEE) to control for covariates and accommodate clustering by doctor. To evaluate the impact of the customary single imputation technique, we also conducted an analysis of the primary outcome with multiple imputation using the method of chained equations (MICE) [18]. The chained equations approach was chosen for its flexibility in handling variables of varying types (i.e., binary, continuous, count). MICE assumes that missing data are missing at random (MAR). That is, the probability of a value being missing may be dependent on observed data (i.e., variables included in the imputation) but not on unobserved data. Twenty imputations were generated using the following variables: intervention; age; gender; ethnicity; race; insurance; number of cigarettes smoked per day at baseline; baseline heaviness of smoking index; baseline use of drugs or alcohol; baseline participant belief that s/he has a tobacco-related illness; baseline participant belief that their hospitalization made worse by smoking; baseline readiness to quit; and self-reported abstinence at 1, 6, and 12 months. Results of the 20 imputations were then summarized using the methods described by Schafer and Graham [19].

Categorical secondary outcomes were compared using chi-square analysis. Cigarettes smoked per day were compared using a repeated measures linear mixed model. Fixed effects in the model included intervention, time (1 month, 6 months, 12 months), intervention by time interaction, and baseline number of cigarettes smoked per day. A random effect was included for doctor, and an unstructured covariance pattern was used to accommodate serial correlation between repeated assessments.

Lastly, we conducted an analysis of potential mediators and moderators of the E-STOPS intervention for the primary outcome, testing pre-specified covariates. For moderators, these included the heaviness of smoking index, readiness to quit, whether the participant believed s/he had a tobacco-related illness, and concurrent self-reported use of drugs or alcohol. For mediators, these included use of nicotine replacement therapies or the state smokers’ quitline. Moderators were evaluated by adding the interaction of the moderator variable with treatment in the GEE described above. Mediators were evaluated by the method described by Mackinnon and Dwyer [20].

## Results

**Fig. 2:**
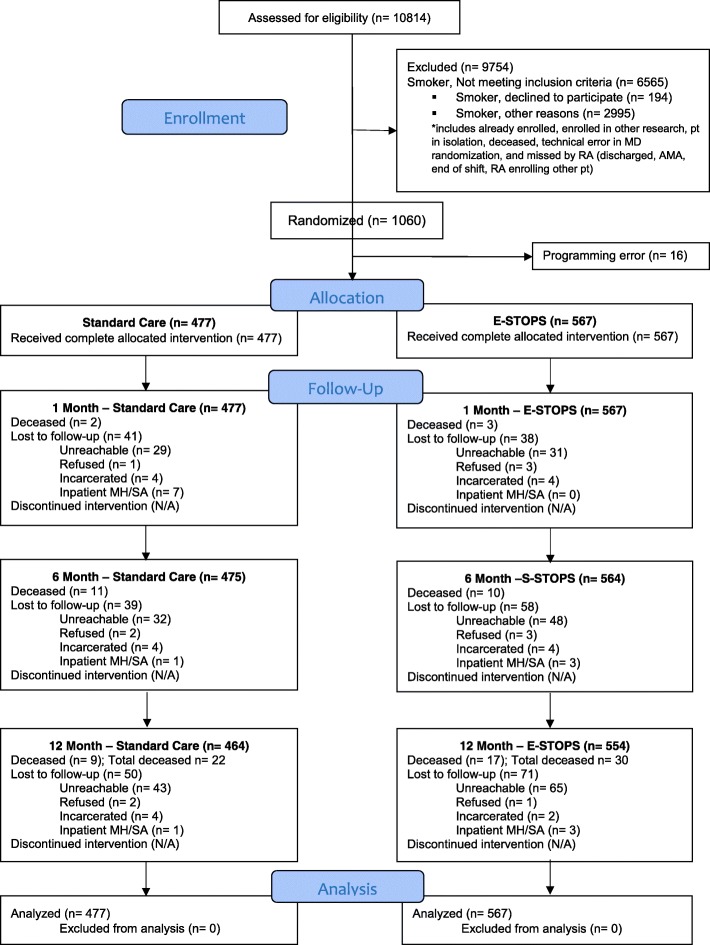
Flow of patients through the trial. The CONSORT diagram shows the flow of patients assessed for eligibility, enrolled, randomized, and analyzed in the trial

The study was conducted from August 2013 to September 2015. A total of 254 physicians were enrolled, including 44 hospitalists, 180 internal medicine residents, and 30 emergency medicine residents. Of all 254 physicians, 126 were randomized to E-STOPS, 128 to control. Figure 2 shows the flow of subjects through the trial.

Of the 9754 patients excluded from the trial, the most common reasons for exclusion were the following: smoking fewer than 5 cigarettes/day (1264), living outside New Haven County (943), having an active psychiatric problem or being unable to provide consent (1292), and unable to be approached by the RA because the patient was not in the hospital room at the time of assessment (1229).

Nearly all contacted physicians agreed to enroll in the trial. More than 90% of residents enrolled. About two thirds of the hospitalists enrolled (non-enrolled hospitalists generally did not attend the staff meetings where the study was discussed; none who attended the meetings declined to give consent).

Table 1 shows the demographic characteristics of the patient participants. The baseline characteristics of the treatment arms were comparable with respect to sociodemographic variables, daily cigarette consumption, and other smoking variables. That more participants are in the intervention condition than the control is incidental; randomization was by physician, not patient.

**Table 1:** Baseline patient characteristics

Variable	Control (*N* = 477)	E-STOPS (*N* = 567)
Age, mean, years (SD)	49.3 (12.6)	49.3 (11.7)
Sex, no. male (%)	239 (50.1)	281 (49.6)
Race/ethnicity, *N* (%)		
White, non-Hispanic	261 (54.7)	327 (57.7)
African-American, non-Hispanic	154 (32.3)	163 (28.8)
Asian/other, non-Hispanic	11 (2.3)	16 (2.8)
Hispanic	51 (10.7)	61 (10.8)
Insurance		
Self-pay	28 (5.9)	31 (5.5)
Medicaid only	240 (50.3)	298 (52.6)
Medicare only	60 (12.6)	60 (10.6)
Medicaid and Medicare	44 (9.2)	66 (11.6)
Private	101 (21.2)	105 (18.5)
Other	6 (0.8)	7 (1.2)
PHQ 9 depression score, median (IQR)	8 (4, 13)	8 (4, 13)
Rapid alcohol screen (+), *N* (%)	144 (30.2)	189 (33.3)
Rapid drug screen (+), *N* (%)	74 (15.5)	108 (19.1)
Cigarettes/day, median, IQR	10.0 (8, 20)	10.5 (8, 20)
Heavy smoking index ≥ 4, *N* (%)	208 (43.6)	210 (37.0)
Subject believes ED visit related to tobacco, *N* (%)	226 (47.4)	288 (50.8)
Subject believes medical illness related to tobacco, *N* (%)	265 (55.6)	336 (59.3)

Notably, there were numerous statistically significant differences in implementation endpoints, as shown in Table 2 and reported previously [8]. Specifically, patients treated by physicians randomized to E-STOPS were more likely to have been prescribed nicotine replacement therapy while hospitalized, to have been referred to the state quitline, and to have “Tobacco Use Disorder” added to the EHR’s problem list.

**Table 2:** Utilization of tobacco order set functions, August 2013–March 2016 (reproduced from Bernstein et al., 2017) [8]

Function	E-STOPS (*N* = 5391 patients)	Control (*N* = 5548 patients)	*p* value
Medications ordered, *N* (%)	1827 (34%)	1591 (29%)	< 0.0001
Tobacco use disorder added to problem list, *N* (%)	2245 (42%)	122 (2%)	< 0.0001
Referral made to quitline, *N* (%)	1584 (29%)	0 (0%)*	< 0.0001
Email sent to primary care provider, *N* (%)	5375 (99%)	N/A	N/A

*Automated capture of these endpoints in the control arm was not possible. However, review of data with quitline personnel indicate that no referrals or emails were sent

Table 3 shows unadjusted self-reported abstinence rates at 1, 6, and 12 months and biochemically confirmed abstinence at 12 months. The latter is the primary effectiveness endpoint. There are no statistically significant differences in tobacco abstinence, self-reported or biochemically confirmed, at any study time point.

**Table 3:** Primary and secondary endpoints at 12 months (unadjusted)*

Variable	E-STOPS (*N* = 567)	Control (*N* = 477)	Odds ratio or difference between groups (95% CI)
7-day abstinence, biochemically verified, *N* (%)	65 (11.5)	53 (11.1)	1.04 (0.70, 1.52)
24 h quit attempt since ED visit, *N* (%)	322 (56.8)	256 (53.7)	1.13 (0.89, 1.45)
7-day abstinence at 1 month, self-report, *N* (%)	87 (15.3)	72 (15.1)	1.02 (0.73, 1.43)
7-day abstinence at 6 months, self-report, *N* (%)	83 (14.6)	68 (14.3)	1.03 (0.73, 1.46)
7-day abstinence at 12 months, self-report, *N* (%)	86 (15.2)	78 (16.4)	0.91 (0.65, 1.28)
Change in daily cigarette consumption, mean (95% CI)	−5.8 (− 6.5, − 5.2)	−5.9 (− 6.6, − 5.2)	0.07 (− 0.9, 1.1)
Used quitline, *N* (%)	13 (2.3)	8 (1.68)	1.38 (0.57, 3.35)

*Missing outcomes imputed as continued smoking

In multivariable GEE analysis of biochemically confirmed abstinence at 12 months (Table 4), E-STOPS did not reach statistical significance. After adjustment, the proportion of abstinence was 11.6% and 11.5% in E-STOPS and control, respectively (OR = 1.01, *p* = 0.94). Increasing age was associated with higher odds of 12-month abstinence. Black race, relative to white, was associated with lower odds of 12-month abstinence. There were no other statistically significant associations in the multivariable model. Of note, analyses, performed with multiple imputation under the assumption that data were missing at random, yielded similar results (19% and 18% biochemically confirmed abstinence in E-STOPS and control, respectively; *p* = 0.75).

**Table 4:** Multivariable GEE model for 12-month biochemically confirmed abstinence

Variable	OR	95% CI	
		Lower	Upper
Intervention (control is referent group)	1.01	0.72	1.42
Male gender (female is referent)	1.18	0.80	1.73
Age	1.02	1.00	1.03
Race/Ethnicity			
White, non-Hispanic	REF	–	–
African-American, non-Hispanic	0.59	0.38	0.91
Asian/other, non-Hispanic	2.22	0.95	5.18
Hispanic	0.95	0.48	1.85

In the moderator analysis, none of the pre-specified covariates (Heaviness of Smoking Index, *p* for interaction with treatment = 0.60; readiness to quit, *p* = 0.48; participant belief that he/she has a tobacco-related illness, *p* = 0.45; or concurrent self-reported use of drugs or alcohol, *p* = 0.83) were found to be statistically significant moderators of the intervention’s impact. Despite the absence of a significant relation between E-STOPS and 12-month biochemically confirmed abstinence, E-STOPS was associated with a greater likelihood of NRT (OR = 1.39, *p* = 0.003) and quitline use (OR = 2.82, *p* < 0.001) while NRT (OR = 1.53, *p* = 0.02) and quitline use (OR = 1.85, *p* = 0.02) were both associated with a greater likelihood of biochemically confirmed abstinence. Tests of the indirect effect demonstrated significant mediation of the relation between E-STOPS and abstinence by both NRT (*p* for mediation = 0.02) and the use of the state smokers’ quitline (*p* = 0.01).

In an exploratory analysis, we examined whether there was a dose-response relationship between the number of E-STOPS components ordered for individual participants and their odds of quitting. To do this, we partitioned the providers into quartiles, based on patterns of use, and examined the participants treated by these providers. In other words, the first quartile of physicians most frequently ordered combinations of medications, ordered quitline referrals, and placed “Tobacco Use Disorder” in the problem list; the fourth quartile ordered these items least often.

The proportions of smokers biochemically confirmed abstinent at 12 months in the first, second, third, and fourth quartiles of provider use of E-STOPS were, respectively, 13.0%, 10.6%, 14.3%, and 9.9% (*p* = 0.57, Mantel-Haenszel chi-square), indicating there was no dose-response relationship between treatments received and probability of abstinence. Similar results were obtained for self-reported abstinence at 1 and 6 months.

Results of the contextual analysis are provided in Table 5. In general, numerous factors were identified during study design that suggested broad support throughout the organization for E-STOPS. Primary points of concern from hospital administrators and information technology (IT) leadership were that the implementation should occur after installation of the new electronic health record (EHR). From the clinicians, the chief concern was that E-STOPS facilitate their work, rather than provide additional “clicks” to navigate in the EHR that did not add value to patient care.

**Table 5:** Contextual analysis. Domains adapted from Stange and Glasgow [10].

Domain	Findings	Implications for E-STOPS design and implementation
Relevant theory or participant mental models	Push-pull capacity model for guideline implementation [11]	Provided conceptual model for study and means of framing E-STOPS for various stakeholders
National, state, local public policy	HITECH act encourages adoption of EHRs; tobacco screening, treatment as early publicly reported core measure	Important “push” factors that facilitated framing of intervention to hospital leadership
Pertinent community norms, resources	Primary care access is modest in local community; care often fragmented between hospital, outpatient providers	Use of health IT/EHR designed to facilitate communication between providers
Health care system organization, payment systems, IT, other support systems	IT reports to finance; new EHR installed near planned launch of E-STOPS need to address potential return on investment for tobacco treatment, re: pay-for-performance and public reporting of core measures; compliance with CMS, Joint Commission mandates	Need to address potential return on investment for tobacco treatment, re: pay-for-performance and public reporting of core measures; compliance with CMS, Joint Commission mandates
Practice culture, staffing	Physicians, nurses want to treat tobacco dependence; may have limited skills, knowledge, resources to do so	E-STOPS designed to minimize provider workload, provide choice, but make treatment the default choice.
Patient populations, subgroups	Many adult smokers admitted to hospital; hospitalization as period of enforced abstinence, “teachable moment” for tobacco	E-STOPS limited to inpatient units on medical services, to capitalize on “teachable moment”.
Relevant historical factors, recent events	Steady decline in prevalence of smoking, but undertreatment still common in healthcare settings; growth of value-based performance models	Used to provide rationale for E-STOPS to physicians, nurses, administrators
Culture, motivations surrounding monitoring, evaluation	Physicians want to treat smokers; some concerns about added workload, role of hospital-based personnel in treating tobacco dependence; concerns about performance assessment	Physicians assured that feedback was confidential, would not be shared with supervisors.

## Discussion

Smokers are frequently admitted to the hospital. Because hospitals are smoke-free, hospitalization represents an opportunity to begin treatment for tobacco dependence and continue it after discharge, when many smokers relapse [21]. However, according to a Cochrane review, tobacco use disorder treatment needs to continue for at least 30 days after discharge to result in long-term cessation [4].

The components included in the E-STOPS decision prompt and order set were chosen for their well-documented effectiveness and presumed ability to leverage treatment for tobacco dependence for at least 30 days after discharge. Specifically, active quitline referral, notification of the primary care provider, and populating the problem list with “Tobacco Use Disorder” were all expected to lead to ongoing treatment. The medication orders, while evidence-based, were limited to prescription of NRT and bupropion during the hospitalization itself. Physicians could prescribe tobacco use disorder medications at discharge, but for technical reasons that was not prompted by E-STOPS.

Although the E-STOPS prompt and order set resulted in a dramatic, sustained increase in the processes of care of tobacco dependence, they did not lead to more tobacco quits than control. Thus, given this was a Type I effectiveness-implementation hybrid design (in which clinical effectiveness is the primary endpoint), E-STOPS was not successful, even though important improvements were seen, physician behaviors regarding the implementation of tobacco dependence treatment. The reasons for this are likely multifactorial.

First, hospitalization itself is a “teachable moment.” Smokers admitted to hospitals often are abstinent from tobacco after discharge, simply because of fear and concern related to the acute health event [22]. Thus, the abstinence rate in the control arm was higher than we initially expected, as often happens in studies of behavioral interventions [23].

Second, the primary endpoint was measured relatively distal to the inpatient intervention. Even though we selected interventions that would, presumably, continue after the discharge home, most of them would not have continued for a full year. This would include the medications that the physician may have prescribed during the hospitalization and the quitline services. The one-year endpoint was selected as primary, rather than the one- or six-month assessments, to provide a standard measure of tobacco abstinence. That said, even those earlier waves of assessment, as recently as one month after hospitalization, showed no difference in abstinence rates, indicating that E-STOPS was not clinically effective.

Finally, the absence of a dose-response relationship between the number of E-STOPS components used and probability of abstinence is disappointing. Again, this likely reflects the inability of individual components to extend treatment after hospital discharge in a clinically meaningful way. This may reflect inadequate engagement with primary care providers in the E-STOPS effort. PCPs received an email message embedded in the EHR itself but were not offered decision support for tobacco dependence treatment embedded in the outpatient modules of the chart.

After follow-up was completed, we conducted semi-structured interviews with 21 physicians randomized to E-STOPS, to better understand facilitators and barriers to use. The sample was evenly divided between high- and low-utilizers of E-STOPS. Using thematic analysis and the constant comparative method, assisted by ATLAS.ti (version 7.1.7), three themes emerged addressing the inpatient environment, prescriber attitudes and beliefs, and information needs. Overall, participants were pleased with E-STOPS but had specific suggestions for improvements regarding the timing of the intervention, suppression logic, and additional decision support and training. A few had concerns about the clinical appropriateness of beginning treatment for tobacco dependence during a hospitalization and the proper role of the inpatient team in that treatment.

These concerns about workflow and timing also accounted for the active deselection of certain E-STOPS functions by physicians. Approximately 70% of E-STOPS firings led to active deselection of the e-referral to the smokers’ quitline; nearly 60% of firings led to active deselection of adding “Tobacco Use Disorder” to the problem list. In the qualitative work, physicians that at the time E-STOPS fired had not yet had the opportunity to interview and examine the patient and so were unsure whether prescription of NRT was indicated or whether the patient would be receptive to engaging with the quitline. In addition, physicians learned that in order to stop E-STOPS from firing, they had to first deselect the pre-ordered functions before declining to accept the best practice alert. This appears to have been the most likely reason for the relative underuse of the functions addressing quitline referral and population of the problem list.

In addition, we should note that we were not able to access information regarding physician’s baseline performance regarding ordering tobacco treatment medications, referring to the quitline, and populating the problem list with tobacco-related diagnoses. The healthcare system installed a new EHR shortly before study onset, and limited patient-level data were migrated over from the legacy system.

The results of the mediator/moderator analysis are of interest. Given the near-identical performance of E-STOPS and treatment as usual on abstinence rates, it is perhaps expected that no baseline covariates were found to moderate the intervention. Ordinarily, one would expect the prescription of NRT to mediate tobacco abstinence. However, the NRT prescribed in this study was administered only during the hospitalization itself. Again, E-STOPS was not able to prompt providers to prescribe NRT (or varenicline) at discharge. Had it done so, a mediational effect might have been seen.

The quitline, on the other hand, was a mediator. Given the Cochrane review’s finding that efficacious interventions begun during hospitalization should continue for at least 30 days after discharge, this makes sense. Quitline services can be delivered for 30 or more days after an intervention, particularly if the individual signs up for the quitline’s 5-call program.

## Limitations

The study was conducted at a single academic medical center in the northeastern U.S. Results in other centers or regions might differ. However, our patient population is quite diverse, and our medical house staff and physicians trained at medical schools and hospitals across the country. Thus, important regional differences in care are unlikely. Of note, most physicians in the study were residents in training, rather than board-certified or board-prepared attendings. This reflects the realities of clinical care in academic medical centers, where much front-line care is delivered by physicians in training, even if under the supervision of attendings. It is possible that attending physicians might have delivered more thorough treatment for smoking. However, we did not see a difference in cessation rates among patients treated by hospitalists (who are attendings) and those treated by residents.

We did not actively seek to engage primary care providers in this study. Our program focused on inpatient interventions and providers. Our hope was that PCPs would, based on the “in-basket” messages and updated problem lists, provide ongoing tobacco use disorder treatment for smokers receiving the intervention. However, we did not assess PCP behavior, including medication prescribing, as this was a hospital-based study. Most, but not all, PCPs shared the same EHR as our hospital, and we lacked the resources to contact them individually.

An additional limitation is that E-STOPS did not include decision support to encourage providers to prescribe tobacco treatment medications to patients at the time of discharge from the hospital. We considered this but were concerned about potential contamination caused by firing of prompts for discharge providers in the E-STOPS arm, who were now treating patients initially cared for by physicians in the control arm. This latter scenario happened regularly, insofar as changes in physician teams, night float coverage, and duty hour restrictions occur routinely in academic medical centers. It is possible that the more routine provision of prescriptions for tobacco treatment medications at discharge would have helped improve short-term abstinence rates.

In addition, E-STOPS did not permit the prescribing of varenicline, a highly effective medication for tobacco dependence treatment. Varenicline is not part of the inpatient formulary in our hospital. This may have had some modest effect on clinical outcomes.

Another potential limitation of E-STOPS was the decision to limit its access to physicians, rather than including nurses, midlevel providers, and other allied healthcare personnel such as respiratory therapists and pharmacists. Again, our intent was to first assess its use by providers with frontline clinical responsibility for patients. Insofar as team-based approaches to tobacco dependence treatment can be more efficacious, future iterations of E-STOPS may do this. An important consideration in a team-based electronic tool would be to develop additional strategies to prevent contamination of control participants by treatment by E-STOPS team members.

One approach to overcome most of these limitations would be to conduct a cluster-randomized trial with more hospitals, using the hospital as the unit of randomization. That way, all providers in a given hospital could be randomized to E-STOPS or control and contamination would not occur. Once we can demonstrate the efficacy of E-STOPS at our institution, we will consider a multicenter study. In addition, future iterations of E-STOPS implementation will seek to enhance engagement of PCPs, to increase the likelihood that smokers will have continued tobacco treatment after hospital discharge.

## Conclusions

Electronic health records can be configured to enhance the treatment of tobacco dependence among hospitalized smokers and can extend treatment beyond the time of discharge. However, whether these EMR-embedded interventions can result in sustained tobacco abstinence is unclear. The initial configuration of E-STOPS did not improve cessation rates beyond that of usual care. Future work will focus on enhancing the efficacy of E-STOPS by varying the timing and frequency of its firing, prompting providers to order medications at discharge, and enhancing the linkages between inpatient and ambulatory treatment for tobacco dependence.
